# G protein βγ translocation to the Golgi apparatus activates MAPK *via* p110γ-p101 heterodimers

**DOI:** 10.1016/j.jbc.2021.100325

**Published:** 2021-01-23

**Authors:** Mostafa Khater, Zhe Wei, Xin Xu, Wei Huang, Bal L. Lokeshwar, Nevin A. Lambert, Guangyu Wu

**Affiliations:** 1 Department of Pharmacology and Toxicology, Medical College of Georgia, Augusta University, Augusta, Georgia, USA; 2 Georgia Cancer Center, Augusta University, Augusta, Georgia, USA

**Keywords:** G protein–coupled receptor, CXCR4, G protein, Gβγ, translocation, Golgi, signaling, MAPK, ERK1/2, PI3Kγ, α2-AR, α2-adrenergic receptor, BFA, brefeldin A, EGF, epidermal growth factor, ERK1/2, extracellular signal-regulated kinases 1 and 2, FKBP, FK506-binding protein, FRB, FKBP-rapamycin binding domain, GA, Golgi apparatus, GPCR, G protein-coupled receptor, HEK293, human embryonic kidney 293, IGF, insulin-like growth factor 1, MAPK, mitogen-activated protein kinase, PI3Kγ, phosphoinositide 3-kinase γ, PM, plasma membrane, PTX, pertussis toxin, RTK, receptor tyrosine kinases, sgRNA, single guide RNA

## Abstract

The Golgi apparatus (GA) is a cellular organelle that plays a critical role in the processing of proteins for secretion. Activation of G protein–coupled receptors at the plasma membrane (PM) induces the translocation of G protein βγ dimers to the GA. However, the functional significance of this translocation is largely unknown. Here, we study PM-GA translocation of all 12 Gγ subunits in response to chemokine receptor CXCR4 activation and demonstrate that Gγ9 is a unique Golgi-translocating Gγ subunit. CRISPR-Cas9–mediated knockout of Gγ9 abolishes activation of extracellular signal-regulated kinase 1 and 2 (ERK1/2), two members of the mitogen-activated protein kinase family, by CXCR4. We show that chemically induced recruitment to the GA of Gβγ dimers containing different Gγ subunits activates ERK1/2, whereas recruitment to the PM is ineffective. We also demonstrate that pharmacological inhibition of phosphoinositide 3-kinase γ (PI3Kγ) and depletion of its subunits p110γ and p101 abrogate ERK1/2 activation by CXCR4 and Gβγ recruitment to the GA. Knockout of either Gγ9 or PI3Kγ significantly suppresses prostate cancer PC3 cell migration, invasion, and metastasis. Collectively, our data demonstrate a novel function for Gβγ translocation to the GA, *via* activating PI3Kγ heterodimers p110γ-p101, to spatiotemporally regulate mitogen-activated protein kinase activation by G protein–coupled receptors and ultimately control tumor progression.

G protein–coupled receptors (GPCRs) modulate a wide variety of cell functions through activating cognate heterotrimeric G proteins, arrestins, and other signaling molecules (1). In the classical GPCR signaling system, GPCRs at the plasma membrane (PM), once activated by hormones or neurotransmitters, function as guanine nucleotide exchange factors to enhance the exchange of GDP for GTP from Gα subunits, leading to the dissociation of active GTP-bound Gα and free Gβγ dimers, which can separately activate downstream effectors, such as adenylyl cyclases, phospholipases, mitogen-activated protein kinases (MAPKs), phosphoinositide 3-kinases (PI3Ks), and ion channels (2, 3, 4). In recent years, several studies have demonstrated that, after activation by GPCRs at the PM, some Gβγ dimers can translocate from the PM to intracellular organelles, including the Golgi apparatus (GA), likely *via* passive diffusion, and that the efficiency of translocation is determined by Gγ anchoring to the PM, as well as Gγ interaction with the receptors (5, 6, 7, 8, 9, 10). Although the Golgi-localized Gβγ complex can activate phospholipase C (11, 12) and protein kinase D (13, 14) and regulate post-Golgi trafficking (14, 15, 16), Golgi structure (13, 16, 17), insulin secretion (17), and cardiomyocyte hypertrophic growth (11), the physiological and pathophysiological functions of Gβγ translocation from the PM to the GA are still largely undefined.

The MAPKs extracellular signal-regulated kinases 1 and 2 (ERK1/2) and PI3Ks are crucially involved in many fundamental cellular processes and are directly associated with the pathogenesis of human diseases, particularly cancer. It is known that almost all GPCRs can activate the MAPK Raf-MEK-ERK1/2 pathway. However, ERK1/2 activation by different GPCRs or the same GPCR in different cell types may be mediated through different biochemical pathways, involving distinct signaling molecules, such as Gβγ subunits, arrestins, small GTPases, receptor tyrosine kinases (RTKs), and protein kinases (18, 19, 20, 21, 22, 23, 24, 25, 26, 27, 28, 29). Although these complex activation mechanisms have been studied extensively and arrestins have been shown to function as scaffolds in ERK1/2 activation on the endosomal compartment, the spatial aspects of ERK1/2 activation by GPCRs remain poorly explored and signal initiation by G proteins is generally considered to occur at the PM (23, 27, 28, 30).

PI3Ks are a family of lipid kinases that specifically phosphorylate the inositol moiety of phospholipids at the 3’ position and can be divided into three classes based on their sequence homology and substrate specificity. Class I PI3Ks include PI3Kα, β, γ, and δ isoforms, which are heterodimers consisting of a catalytic subunit and a regulatory subunit, and the PI3Kγ catalytic subunit p110γ can form a complex with the regulatory subunit p101 or p87. It has been known that the cell surface GPCRs can activate PI3Kβ, γ, and δ isoforms and Gβγ dimers activate PI3Kβ and γ isoforms. PI3Kγ activation by Gβγ has been extensively studied, and these studies have shown that Gβγ can directly interact with both p110γ and p101 (31, 32, 33).

Over the past decades, multiple signaling cascades have been defined to contribute to prostate tumorigenesis. Among these cascades, the ERK1/2 and PI3K pathways are hyperactivated in prostate cancer and enhanced activation of ERK1/2 and PI3Ks is correlated with disease progression, androgen independence, and poor prognosis (33, 34, 35, 36). However, the molecular mechanisms responsible for the hyperactivation of the ERK1/2 and PI3K pathways in prostate cancer remain poorly understood. Here we study the function of Gβγ translocation to the GA in ERK1/2 activation by the chemokine receptor CXCR4 in human androgen-insensitive prostate cancer (DU145 and PC3) cells and human embryonic kidney 293 (HEK293) cells. CXCR4 is a crucial factor involved in bone metastasis of prostate cancer and CXCR4 antagonists inhibit prostate cancer growth and metastasis (37, 38). We demonstrate that Gβγ translocation to the GA activates the ERK1/2 pathway through the PI3Kγ heterodimer p110γ-p101. We also show that knockout of the most translocatable Gγ9 subunit and p110γ markedly inhibits prostate cancer cell migration, invasion, and metastasis. These data reveal a novel function of Gβγ translocation to the GA to spatiotemporally regulate MAPK activation by GPCRs and control tumor progression.

## Results

### Characterization of Gβγ translocation to the GA in response to CXCR4 activation

**Figure 1:**
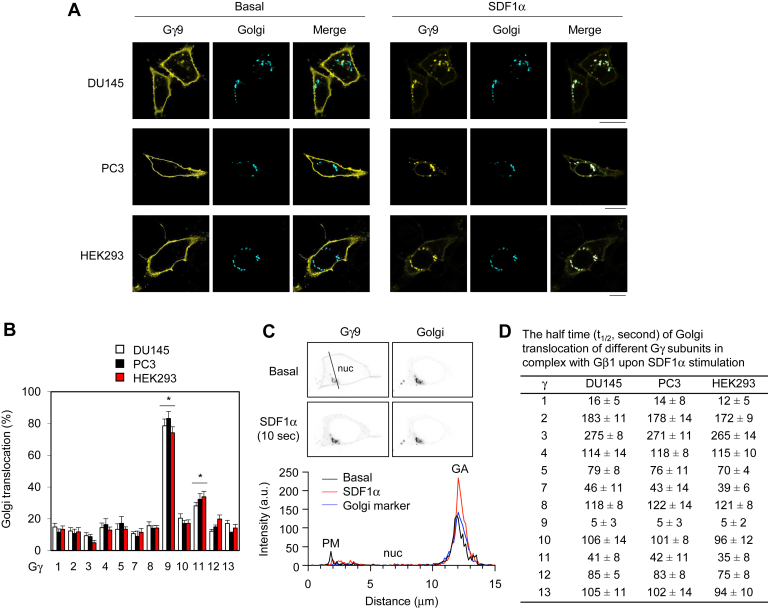
**Gβγ translocation from the PM to the GA in DU145, PC3, and HEK293 cells.***A*, Gγ9 translocation from the PM to the GA. The cells were cultured on 6-well dishes and transfected with YFP-Gγ9, Gβ1, Gαi1, and pmTurquoise2-Golgi (500 ng each). After starvation for 48 h, the cells were stimulated with SDF1α at 1 μg/ml. The images shown are obtained after stimulation for 10 s. *B*, quantification of the translocation of different Gγ subunits in complex with Gβ1 in response to SDF1α stimulation. The cells were transfected with individual YFP-Gγ, Gβ1, Gαi1, and pmTurquoise2-Golgi and stimulated as in *A*. The YFP signal at the GA before and after SDF1α stimulation was measured as shown in Fig. S1A. The increase in the YFP signal at the GA after SDF1α stimulation was expressed as the translocation of Gβγ to the GA. ∗*p* < 0.05 *versus* Gγ3. *C*, relative expression of YFP-Gγ9 at the PM, nucleus (nuc), and GA before and after SDF1α stimulation for 10 s in a representative DU145 cell based on the line scan analysis. *D*, the half time (t_1/2_) of Gγ translocation from the PM to the GA after SDF1α stimulation. The images shown in *A* and *C* are representatives of five to eight experiments. The quantitative data are presented as means ± SD (n = 5–8). The scale bars represent 10 μm. GA, Golgi apparatus; PM, plasma membrane.

Individual YFP-tagged Gγ subunits were expressed together with Gβ1, a commonly expressed Gβ subunit, Gαi1 subunit, and the Golgi marker pmTurquoise2-Golgi in DU145, PC3, and HEK293 cells. Gβγ translocation to the GA in response to CXCR4 activation by stimulation with stromal cell–derived factor 1α (SDF1α) was measured by the increase in total YFP signal at the GA by confocal microcopy in live cells (Fig. S1*A*). All 12 Gγ subunits were mainly expressed at the PM in unstimulated cells, and SDF1α stimulation induced Gγ translocation from the PM to intracellular compartments at different magnitudes and rates. However, translocation of Gγ9 to the GA was dramatically more efficient than that of any other Gγ subunit (Fig. 1*A* and Fig. S1*B*). The total Gγ9 signal at the GA increased by approximately 80% (Fig. 1*B*). The quantification of relative expression of Gγ9 at the PM, nucleus, and GA showed that SDF1α stimulation markedly reduced Gγ9 expression at the PM and increased Gγ9 expression at the GA (Fig. 1*C*). Translocation of Gγ9 from the PM to the GA occurred with a half time (t_1/2_) of about 5 s (Fig. 1*D*). The closest Gγ subunit to Gγ9 was Gγ11, which GA translocation increased by only 30%. In contrast, Gγ3 showed the least translocation (<10%) (Fig. 1*B*). Across Gγ subunits, the kinetics and extent of translocation were similar in DU145, PC3, and HEK293 cells (Fig. 1, *B* and *D*). These data indicate that Gγ9 is a unique Golgi-translocating Gγ subunit.

### Gβγ translocation to the GA is required for ERK1/2 activation by CXCR4

**Figure 2:**
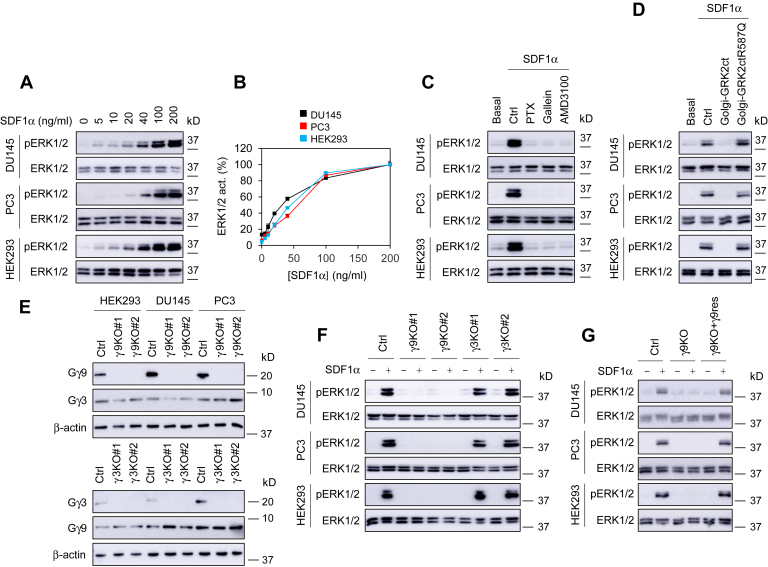
**Gγ9 subunit is required for ERK1/2 activation by SDF1α.***A*, SDF1α dose dependently activated ERK1/2. The cells were cultured on 6-well dishes. After starvation for 48 h, the cells were stimulated with different concentrations of SDF1α for 5 min. *B*, quantitative data shown in *A*. *C*, effect of PTX, gallein, and AMD3100 on ERK1/2 activation by SDF1α. The cells were incubated with PTX (100 ng/ml for 16 h), gallein (10 μM for 30 min), and AMD3100 (100 μM for 1 h) before SDF1α stimulation (200 ng/ml for 5 min). *D*, effect of Golgi-targeting GRK2ct (Golgi-GRK2ct) on ERK1/2 activation by SDF1α. The cells were transfected with Golgi-GRK2ct or its mutant GRK2ctR587Q and then stimulated with SDF1α. *E*, expression of endogenous Gγ3 and Gγ9 in CRISPR-Cas9–mediated Gγ9 (*left panel*) or Gγ3 (*right panel*) knockout cells by Western blotting using Gγ-specific antibodies. *F*, ERK1/2 activation in Gγ3 and Gγ9 knockout cells in response to SDF1α stimulation. *G*, rescue of ERK1/2 activation in response to SDF1α stimulation by transient expression of sgRNA-resistant Gγ9 (γ9res) in Gγ9 knockout PC3 cells. The quantitative data are presented as means ± SD (n = 3). The Western blots shown in each panel are representatives of at least three experiments. PTX, pertussis toxin.

To study the function of Gβγ translocation, we focused on ERK1/2 activation. SDF1α strongly activated ERK1/2 in a dose-dependent manner, and the EC_50_ values were 3.5 ± 0.09, 6.3 ± 0.2, and 5.8 ± 0.03 nM (n = 3) in DU145, PC3, and HEK293 cells, respectively (Fig. 2, *A* and *B*). ERK1/2 activation by SDF1α was completely blocked by treatments with pertussis toxin (PTX), the Gβγ inhibitor gallein (39), and the selective CXCR4 antagonist AMD3100 (Fig. 2*C*). Transient expression of Golgi-GRK2ct, which sequesters free Gβγ dimers at the GA, abolished ERK1/2 activation by SDF1α. In contrast, expression of the Golgi-GRK2ctR587Q mutant, which does not bind Gβγ, had no effect on ERK/2 activation (Fig. 2*D* and Fig. S2). These data suggest an important role of Golgi-localized Gβγ in ERK1/2 activation by CXCR4.

In order to discriminate the roles of translocating and nontranslocating Gγ subunits in MAPK activation, we used the CRISPR-Cas9 genome editing system to selectively knock out Gγ9 or Gγ3 in DU145, PC3, and HEK293 cells. Gγ9 and Gγ3 knockout cells were verified by Western blotting using Gγ-specific antibodies (Fig. 2*E*). Gγ9 knockout did not affect Gγ3 expression, and vice versa (Fig. 2*E*). Gγ9 and Gγ3 knockout cells were also confirmed by the respective depletion of transiently expressed YFP-Gγ9 and YFP-Gγ3, without affecting YFP-Gγ2 expression (Fig. S3*A*). Gγ9 knockout in all three cells almost completely inhibited ERK1/2 activation by SDF1α, whereas Gγ3 knockout did not have a clear effect (Fig. 2*F*). Gγ9 knockout abolished ERK1/2 activation after SDF1α stimulation at all time points tested in PC3 cells (Fig. S3, *B* and *C*). Furthermore, transient expression of single guide RNA (sgRNA)-resistant Gγ9 successfully rescued ERK1/2 activation by SDF1α in Gγ9 knockout cells (Fig. 2*G* and Fig. S3*D*). These data strongly indicate that the normal expression of endogenous Gγ9 is essential for ERK1/2 activation by CXCR4.

### Constitutive targeting of Gβγ to the GA directly activates ERK1/2

**Figure 3:**
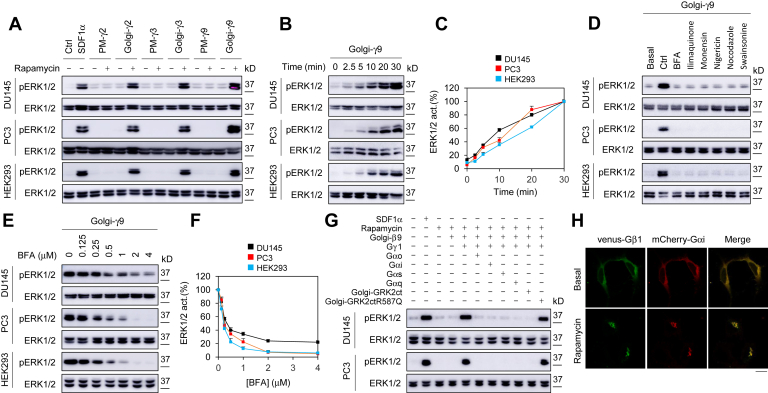
**Inducible expression of Gβγ at the GA directly activates ERK1/2.***A*, inducible expression of Gγ2, Gγ3, and Gγ9 at the GA, but not at the PM, activated ERK1/2. The cells were cultured on 6-well dishes, transfected with individual FRB-Gγ and Gβ1, together with Golgi-Gγ or PM-Gγ (500 ng each), and then induced with rapamycin at 1 μM for 30 min. SDF1α simulation (200 ng/ml for 5 min) was used as a control. *B*, time courses of ERK1/2 activation by inducible expression of Gγ9 at the GA. *C*, quantitative data shown in *B*. *D*, effect of Golgi disruptors on Gγ9-mediated ERK1/2 activation. The cells transfected with FRB-Gγ9, Gβ1, and Golgi-FKBP were treated with BFA (3 μM), ilimaquinone (10 μM), monensin (5 μM), nigericin (2 μM), nocodazole (10 μM), and swainsonine (5 μM) for 40 min before incubation with rapamycin for 30 min to induce Gβγ translocation. *E*, dose-dependent action of BFA on Golgi-Gγ9-induced ERK1/2 activation. The cells were transfected with FRB-Gγ9, Gβ1, and Golgi-FKBP and then treated with BFA at different concentrations (0–4 μM) for 40 min before incubation with rapamycin for 30 min to induce Gβγ translocation. *F*, quantitative data shown in *E*. *G*, effect of Gβ, Gα, and GRK2ct on ERK1/2 activation by Golgi-Gγ9. The cells were transfected with FRB-Gγ9 and Golgi-FKBP with or without cotransfection with Gβ1, different Gα subunits, Golgi-GRK2ct, or GRK2ctR587Q (500 ng each) and then incubated with rapamycin for 30 min. ERK1/2 activation by SDF1α (200 ng/ml for 5 min) was used as a control. *H*, Gβγ translocation in the presence of Gα subunits. PC3 cells were transfected with venus-Gβ1, mCherry-Gαi1, FRB-Gγ9, and Golgi-FKBP (500 ng each) and then treated with rapamycin at 1 μM for 30 min. The quantitative data are presented as means ± SD (n = 3). The Western blots and images shown are representatives of at least three experiments. The scale bar represents 10 μm. BFA, brefeldin A; FRB, FKBP-rapamycin binding; GA, Golgi apparatus; PM, plasma membrane.

To further define the role of Gβγ translocation to the GA in ERK1/2 activation, a rapamycin-inducible translocation system was utilized to specifically recruit Gβγ dimers to either the GA or the PM. In this system, specific targeting peptides (*i.e.*, the mutant KDELr-D193N for GA targeting and amino acids 1–11 of Lyn for PM targeting) were fused to FK506-binding protein (FKBP), while individual cytosolic Gγ subunits were fused to the FKBP-rapamycin binding (FRB) domain. This system has been used previously to recruit Gβγ to the GA and the PM in HeLa cells and cardiomyocytes (11, 14). The system was confirmed by using venus-Gβ1 and Gγ9 (Fig. S4). Of interest, rapamycin-induced recruitment of Gγ2, Gγ3, or Gγ9 to the GA (Golgi-Gγ), each in complex with Gβ1, was able to activate ERK1/2 in DU145, PC3, and HEK293 cells, whereas recruitment to the PM (PM-Gγ) had no obvious effect (Fig. 3*A*). Rapamycin incubation to induce Gγ9 translocation to the GA activated ERK1/2 in a time-dependent fashion (Fig. 3, *B* and *C*). These data suggest that different Gβγ dimers can specifically activate ERK1/2 if they are present at the GA.

To verify if Golgi-Gβγ–mediated activation of ERK1/2 indeed occurred at the GA, we measured the effects of well-known Golgi disruptors, including brefeldin A (BFA), ilimaquinone, monensin, nigericin, nocodazole, and swainsonine, which induce Golgi fragmentation *via* distinct mechanisms (40). Incubation with each of these Golgi disruptors caused Golgi fragmentation as indicated by GalT, a *trans*-Golgi marker (Fig. S5), and abolished ERK1/2 activation induced by Golgi-Gγ9 (Fig. 3*D*). BFA dose dependently inhibited ERK1/2 activation by Golgi-Gγ9 expression, and the IC_50_ values were 0.37 ± 0.03, 0.19 ± 0.05, and 0.18 ± 0.03 μM (n = 3) in DU145, PC3, and HEK293 cells, respectively (Fig. 3, *E* and *F*). These data demonstrate that the integrity of the Golgi structure is required for ERK1/2 activation by Golgi-Gγ. As BFA treatment to disrupt the GA inhibits CXCR4 expression at the PM, likely by interfering its anterograde transport from the endoplasmic reticulum to the cell surface (41), we did not measure the effects of these Golgi disruptors on ERK1/2 activation by SDF1α.

We then determined the effect of different Gα subunits on ERK1/2 activation by Gβγ translocation to the GA. Expression of Gαo, Gαi, Gαs, or Gαq subunits each remarkably attenuated ERK1/2 activation by Golgi-Gγ9 in DU145 and PC3 cells (Fig. 3*G*). In the presence of Gαi subunits, Gβγ normally translocated to the GA. Similar to Gβγ, Gαi also translocated to the GA and strongly colocalized with Gβγ (Fig. 3*H*), suggesting that overexpression of Gα subunits inhibits Gβγ-mediated ERK1/2 activation by forming the inactive Gαβγ heterotrimers on the GA. Similar to SDF1α-mediated ERK1/2 activation, Golgi-Gγ9–induced ERK1/2 activation was inhibited by expression of Golgi-GRK2ct but not Golgi-GRK2ctR587Q mutant (Fig. 3*G*). Furthermore, expression of Gγ9 alone, without coexpression of Gβ1, was unable to activate ERK1/2 (Fig. 3*G*), suggesting a functional unit of Gβγ complex, not just Gγ subunit alone, in ERK1/2 activation.

### ERK1/2 activation by Gβγ translocation to the GA is mediated through the PI3Kγ heterodimer p110γ-p101

**Figure 4:**
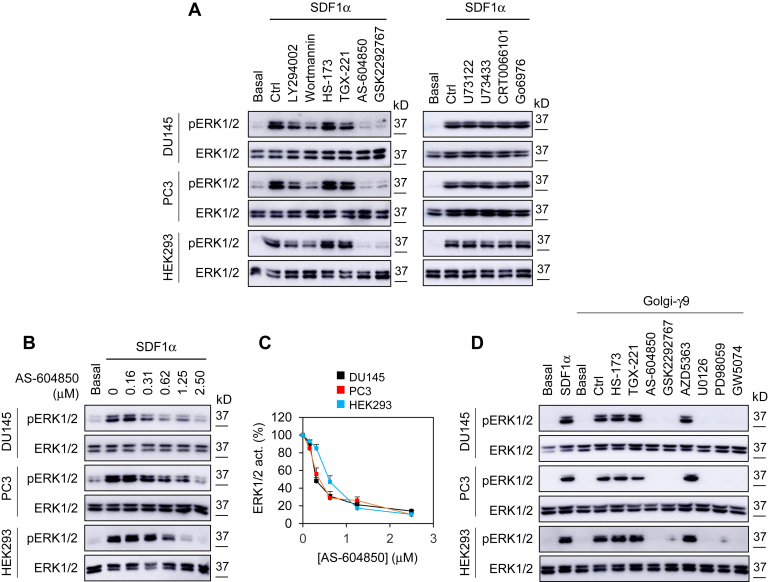
**Effect of pharmacological inhibition of Gβγ downstream effectors on ERK1/2 activation by SDF1α and Golgi-Gγ9.***A*, the cells were incubated with LY294002 (50 μM), wortmannin (10 μM), HS-173 (0.1 μM), TGX-221 (0.5 μM), AS-604850 (2.5 μM), and GSK2292767 (0.5 μM) for 6 h (*left panel*); U73122 (10 μM), U73433 (10 μM), CRT0066101 (5 μM) for 1 h, or Go6976 (1 μM) for 30 min (*right panel*) before stimulation with SDF1α at 200 ng/ml for 5 min. *B*, dose-dependent effect of AS-604850 treatment for 6 h on ERK1/2 activation by SDF1α. *C*, quantitative data shown in *B*. *D*, the cells were transfected with FRB-Gγ9, Gβ1, and Golgi-FKBP (500 ng each) and then incubated with HS-173, TGX-221, AS-604850, and GSK2292767 as in *A*; AZD5363 (1 μM) for 3 h; U0126 (10 μM), PD98059 (50 μM), or GW5074 (10 μM) for 14 h before incubation with rapamycin for 30 min. The quantitative data are presented as means ± SD (n = 3). The Western blots shown in each panel are representatives of at least three experiments.

To elucidate the molecular mechanisms underlying the function of Golgi-localized Gβγ in ERK1/2 activation, we first measured the effects of pharmacological inhibition of well-known Gβγ downstream effectors on ERK1/2 activation by SDF1α. Treatments with LY294002 and wortmannin, two common PI3K inhibitors, partially inhibited ERK1/2 activation by SDF1α. The PI3Kγ inhibitor AS-604850 and the PI3Kδ inhibitor GSK2292767 markedly attenuated ERK1/2 activation, but the PI3Kα inhibitor HS-173, the PI3Kβ inhibitor TGX-221, the phospholipase C inhibitor U73122, U73433 (an inactive derivative of U73122), the protein kinase D inhibitor CRT006610, and the protein kinases C inhibitor Go6976 had no effect (Fig. 4*A*). Inhibition of ERK1/2 activation by AS-604850 was in a dose-dependent fashion, and the IC_50_ values were 0.32 ± 0.02, 0.39 ± 0.05, and 0.59 ± 0.07 μM (n = 3) in DU145, PC3, and HEK293 cells, respectively (Fig. 4, *B* and *C*). ERK1/2 activation induced by recruitment of Gγ9 to the GA was also abrogated by treatments with AS-604850 or GSK2292767, but not HS-173 or TGX-221 (Fig. 4*D*). Of interest, the AKT inhibitor AZD5363 did not alter ERK1/2 activation by Golgi-Gγ9 (Fig. 4*D*), suggesting that ERK1/2 activation by Golgi-localized Gβγ is mediated through an AKT-independent mechanism. As positive controls, treatments with two MEK inhibitors, U0126 and PD98059, and the Raf1 inhibitor GW5074 abolished ERK1/2 activation by Golgi-Gγ9 (Fig. 4*D*).

**Figure 5:**
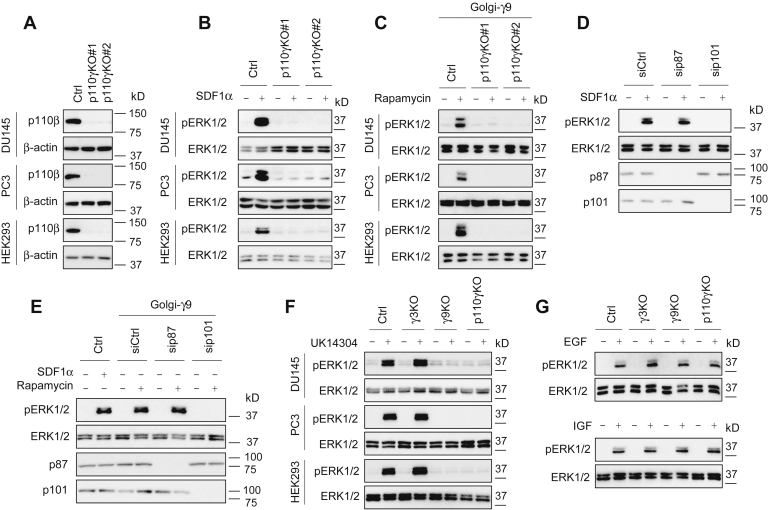
**Depletion of p110γ and p101 abolishes ERK1/2 activation by SDF1α and Golgi-γ9.***A*, expression of p110γ in control and p110γ knockout cells. *B*, ERK1/2 activation by SDF1α at 200 ng/ml for 5 min in control and p110γ knockout cells. *C*, Golgi-γ9-induced ERK1/2 activation in p110γ knockout cells. The cells were transfected with FRB-γ9, Gβ1, and Golgi-FKBP (500 ng each) and then incubated with rapamycin for 30 min. *D*, ERK1/2 activation by SDF1α at 200 ng/ml for 5 min in p87 and p101 knockdown PC3 cells. *E*, Golgi-γ9–induced ERK1/2 activation in p87 and p101 knockdown PC3 cells. *F*, effect of Gγ3, Gγ9, and p110γ knockout on ERK1/2 activation by UK14304 at 1 μM for 5 min. *G*, effect of Gγ3, Gγ9, and p110γ knockout on ERK1/2 activation by EGF at 50 ng/ml for 5 min (*upper panel*), and insulin-like growth factor 1 at 200 ng/ml for 1 h (*lower panel*) in PC3 cells. The Western blots shown in each panel are representatives of at least three experiments.

To further investigate the role of PI3Kγ in ERK1/2 activation, we generated p110γ knockout DU145, PC3, and HEK293 cells using the CRISPR-Cas9 system (Fig. 5*A*). Similar to the results obtained from Gγ9 knockout cells, SDF1α was unable to activate ERK1/2 in p110γ knockout cells (Fig. 5*B*). Furthermore, inducible translocation of Gγ9 to the GA failed to activate ERK1/2 in p110γ knockout cells (Fig. 5*C*). These data demonstrate that ERK1/2 activation by SDF1α and Gβγ translocation to the GA depends on PI3Kγ.

To define the role of regulatory subunits of PI3Kγ in ERK1/2 activation by Gβγ, we determined the effect of siRNA-mediated knockdown of p101 and p87. Similar to p110γ knockout by CRISPR-Cas9, p101 knockdown by siRNA markedly inhibited ERK1/2 activation by SDF1α and Golgi-Gγ9 in PC3 cells, whereas p87 knockdown had no effect (Fig. 5, *D* and *E*). To study if p110γ and p101 were expressed at the GA, p110γ and p101 tagged with GFP or DsRed were transiently expressed together with the Golgi marker pmTurquoise2-Golgi in PC3 cells. As expected, p110γ and p101 were mainly expressed in the cytoplasm and extensively colocalized. Both p110γ and p101 were partially colocalized with the Golgi marker (Fig. S6). These data suggest that p110γ-p101 heterodimers, but not p110γ-p87 heterodimers, mediate ERK1/2 activation by Gβγ on the GA.

To determine if Gβγ and PI3Kγ were also important for ERK1/2 activation by other endogenous GPCRs in DU145, PC3, and HEK293 cells, we examined α_2_-adrenergic receptors (α_2_-ARs). Stimulation with UK14304, an α_2_-AR agonist, markedly activated ERK1/2, which was inhibited by treatments with PTX, gallein, and the α_2_-AR antagonist rauwolscine (Fig. S7*A*). α_2_-AR–mediated ERK1/2 activation was also attenuated by treatments with LY94002, wortmannin, AS-604850, or GSK2292767, but not HS-173 or TGX-221 (Fig. S7*B*). Similar to their effects on ERK1/2 activation by SDF1α, knockout of Gγ9 and p110γ, but not Gγ3, inhibited ERK1/2 activation by UK14304 (Fig. 5*F*). In contrast, Gγ9 and p110γ knockout did not affect ERK1/2 activation by epidermal growth factor (EGF) and insulin-like growth factor 1 (Fig. 5*G* and Fig. S8). These data suggest that Gβγ translocation to the GA may be a common event through which multiple GPCRs converge to activate MAPK *via* PI3Kγ.

### Knockout of Gγ9 and PI3Kγ reduces prostate cancer cell migration, invasion, and metastasis

**Figure 6:**
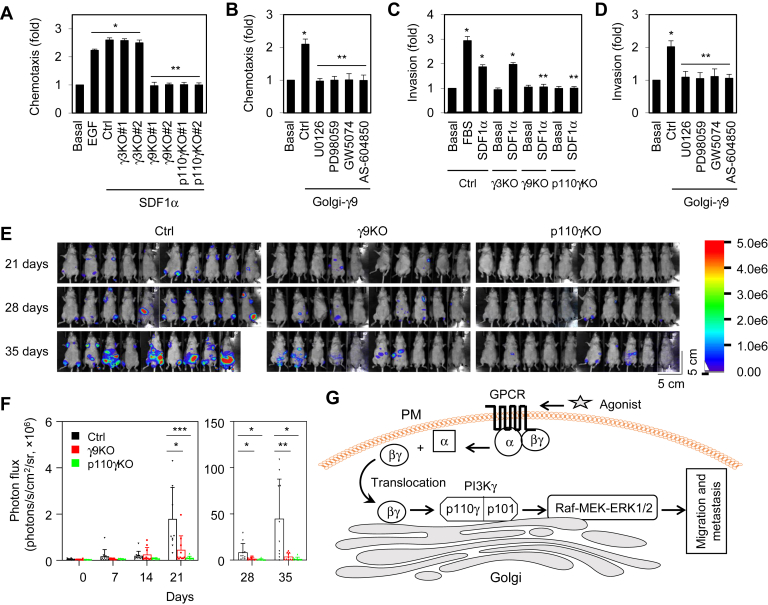
**Effect of Gγ9 and PI3Kγ knockout on PC3 migration and invasion *in vitro* and metastasis *in vivo*.***A*, inhibition of PC3 migration by Gγ9 and p110γ knockout after SDF1α stimulation at 1 μg/ml for 48 h as measured in transwell assays. EGF stimulation (50 ng/ml for 48 h) in control cells was used as a positive control. *B*, enhancement of PC3 migration by Golgi-Gγ9. PC3 cells were transfected with FRB-Gγ9, Gβ1, and Golgi-FKBP (500 ng each) and treated with rapamycin at 1 μM with or without U0126 (10 μM) and PD98059 (50 μM), GW5074 (10 μM), or AS-604850 (2.5 μM) for 48 h. *C*, inhibition of PC3 invasion by Gγ9 and p110γ knockout after SDF1α stimulation at 1 μg/ml for 48 h. FBS (10%) stimulation in control cells was used as a positive control. *D*, PC3 invasion induced by Golgi-Gγ9. PC3 cells were transfected with FRB-Gγ9, Gβ1, and Golgi-FKBP (500 ng each) and treated with rapamycin with or without U0126 and PD98059, GW5074, or AS-604850 as in *B*. *E*, the tumor growth in nude mice inoculated with PC3 cells by intracardiac injection at 21, 28, and 35 days (n = 10 in each group). One mouse died in the control group at 29 days. *F*, quantitative data of tumor growth as measured by bioluminescence imaging of whole animals. *G*, a model depicting the function of Gβγ translocation from the PM to the GA in activation of the ERK1/2 pathway *via* the PI3Kγ heterodimer p110γ-p101 (see text for details). The quantitative data are presented as means ± SD (n = 3 in *A*–*D*; n = 9–10 in *F*). ∗ and ∗∗, *p* < 0.05 *versus* basal and Ctrl, respectively, in *A*–*D*. ∗*p* < 0.05; ∗∗*p* < 0.005; ∗∗∗*p* < 0.001 in *F*. The scale bars represent 5 cm. EGF, epidermal growth factor; FBS, fetal bovine serum.

We next used PC3 cells to determine the effect of Gγ9 and PI3Kγ knockout on cancer cell migration and invasion *in vitro*. In the transwell migration assay, migration of PC3 cells lacking Gγ9 and p110γ in response to SDF1α stimulation was markedly inhibited as compared with control cells. In contrast, Gγ3 knockout PC3 cells migrated normally (Fig. 6*A*). PC3 cell migration was significantly enhanced after rapamycin induction in cells expressing Golgi-Gγ9. The Golgi-Gγ9–induced motility was completely blocked by the ERK1/2 pathway inhibitors U0126, PD98059, and GW5074, as well as the PI3Kγ inhibitor AS-604850 (Fig. 6*B*). Similar to migration, PC3 cell invasion in response to SDF1α stimulation was attenuated in Gγ9 and p110γ knockout cells, but not in Gγ3 knockout cells, as compared with control cells (Fig. 6*C*). Inducible recruitment of Gγ9 to the GA also enhanced PC3 cell invasion, which was blocked by MAPK and PI3Kγ inhibitors (Fig. 6*D*).

Finally, we determined the effect of Gγ9 and PI3Kγ knockout on PC3 metastasis *in vivo*. Gγ9 and p110γ knockout PC3 cells expressing luciferase were injected into the left ventricle of athymic nude mice to allow the cells to disseminate to multiple organs, predominantly lumbar and vertebral bones. Whole-body bioluminescence imaging for luciferase activity and subsequent bioluminescence showed that the overall tumor sizes were dramatically smaller in the group of mice injected with Gγ9 and p110γ knockout cells after 3 weeks as compared with those in the mice injected with control cells (Fig. 6, *E* and *F*). These data demonstrate a crucial role played by Gγ9 and PI3Kγ in prostate tumor metastasis.

## Discussion

In this study, we demonstrate that Gβγ translocation from the PM to the GA is essential for ERK1/2 activation by CXCR4 in three different cells. We have shown that Gβγ dimers that contain Gγ9 are uniquely efficient with respect to translocation to the GA, both in terms of translocation rate and translocation magnitude in DU145, PC3, and HEK293 cells. These results are highly complementary to previous results in other cell types (5, 7, 9, 10, 17). The function of Gβγ translocation to the GA in activating ERK1/2 is strongly supported by three series of experiments that demonstrate that knockout of Gγ9 abolishes ERK1/2 activation by SDF1α, chemically induced translocation of Gγ9 to the GA constitutively activates ERK1/2, and ERK1/2 activation by inducible translocation of Golgi-Gγ9 is completely blocked by Golgi-localized GRK2ct and Golgi disruptors. It is worth noting that, as with Gγ9, recruitment of Gγ2 and Gγ3 on the GA also causes ERK1/2 activation, suggesting that different Gβγ combinations are able to activate ERK1/2 if they are expressed at the GA. However, future studies using Gγ9 mutants defective in GA translocation and defining the ERK1/2 activation signal at the GA may fully clarify the importance of Gβγ translocation in ERK1/2 activation. Since several GPCRs have been shown to promote Gβγ translocation to the GA in different cells (5, 6, 7, 8, 9, 10, 42) and our data have shown that, in addition to CXCR4, α_2_-AR–mediated ERK1/2 activation is also inhibited by Gγ9 knockout, the mechanism we describe here may be commonly used by many GPCRs to activate the ERK1/2 pathway (Fig. 6*G*).

Gβγ is capable of activating many downstream signaling molecules (2, 3). We have demonstrated here that ERK1/2 activation by Golgi-localized Gβγ is mediated through PI3Kγ, specifically its heterodimer p110γ-p101. This became evident as specific pharmacological inhibition of PI3Kγ, CRISPR-Cas9–mediated knockout of catalytic subunit p110γ, and siRNA-mediated knockdown of regulatory subunit p101 abolished ERK1/2 activation by GPCR agonists and translocation of Golgi-Gγ9. This is also supported by our data showing that both p110γ and p101 are partially expressed at the GA and a previous study showing that PI3Ks may be expressed at the GA (43). PI3Kδ may also play a role in Gβγ-mediated ERK1/2 activation, because its inhibition attenuated ERK1/2 activation. Indeed, PI3Kγ and δ can be activated by GPCRs and PI3Kγ can be activated by Gβγ (31, 32, 33). Although a number of previous studies have established the function of Gβγ and PI3Kγ in GPCR-mediated ERK1/2 activation, these studies suggest that the crucial interaction may occur at the PM (18, 19, 20). For example, PI3Kγ was shown to be required for M2-muscarinic receptor- and Gβγ-mediated activation of ERK1/2, likely *via* a typical PM RTK cascade (20). In addition, Gγ3 regulates macrophage migration *via* activating PI3Kγ at the PM, whereas Gγ9 has minimal effect (10). These data suggest that different Gβγ dimers may activate PI3Kγ in distinct subcellular compartments to control different cellular processes, adding to the complexity of Gβγ-mediated signaling and functional regulation. Nevertheless, our data demonstrate a signal transduction pathway in which GPCR activation at the PM induces Gβγ translocation to the GA where it activates the PI3Kγ heterodimer p110γ-p101, leading to the activation of the MAPK pathway (Fig. 6*G*). These data also suggest a novel function of the GA as a signaling organizing compartment in MAPK activation by GPCRs, in which the GA provides a spatial station to compartmentalize the translocation of Gβγ, activation of PI3Kγ, and activation of ERK1/2 pathway (Fig. 6*G*).

Another important finding of the present study is the possible pathophysiological function of Gβγ translocation to the GA and subsequent ERK1/2 activation in prostate tumor progression. Over the past decade, many studies have demonstrated that, similar to RTKs, GPCRs at the PM are involved in the initiation and progression of many different cancer types and significant efforts are currently underway to develop GPCR- and G protein–based drugs for cancer (44, 45). Given the importance of the ERK1/2 pathway in the progression of prostate cancer, the molecules involved in regulation of this pathway are thought to be appealing targets for prostate cancer therapeutics (34, 35). Although multiple genetic mutations in RTKs, Ras, Raf, and MEK cause constitutive activation of the ERK1/2 pathway and drive many types of malignancies (46, 47), patients with prostate cancer frequently do not have these oncogenic mutations. Therefore, extensive efforts are focused on the identification of regulators that control ERK1/2 activation in prostate cancer cells (48). We have shown that Gγ9 is a very strong activator of ERK1/2 at the GA. In addition, both Gγ9 (49) and CXCR4 (38) are highly expressed in prostate cancer cells. As such, enhanced expression and exaggerated activation of GPCRs and G proteins (*e.g.*, CXCR4 and Gγ9) may represent crucial mechanisms responsible for the enhanced activation of the oncogenic ERK1/2 pathway in prostate cancer. As we have demonstrated that knockout of Gγ9 and PI3Kγ markedly suppresses prostate cancer cell migration, invasion, and metastasis, these data, together with previous studies showing the roles of Gβγ in prostate cancer progression (50, 51), imply that Golgi-localized Gβγ, as well as PI3Kγ, may be important targets for prostate cancer therapy.

## Experimental procedures

### Materials

Human SDF1α was purchased from PeproTech; UK14304, GW5074, rapamycin, BFA, ilimaquinone, monensin, nigericin, swainsonine, and LY294002 were from Sigma Aldrich; nocodazole, insulin-like growth factor 1, AMD3100, control siRNA (medium GC), siRNAs targeting to human PI3Kγ regulatory subunits p101 and p87, and antibodies against GFP, phospho-ERK1/2, Gγ9, and β-actin and the PI3Kγ subunits p110γ, p101 and p87 were from Santa Cruz Biotechnology; wortmannin, AS-604850, and GSK2292767 were from ApexBio; TGX-221 and HS-173 were from Adooq Bioscience; U0126 and PD98059 were from Calbiochem; EGF, puromycin, and blasticidin S were from Thermo Fisher Scientific; U-73122 and AZD5363 were from MedChemExpress; U-73433, CRT0066101, and Go6976 were from Cayman Chemical; PTX was from List Biological Laboratories; gallein and rauwolscine were from Tocris Bioscience; D-Luciferin was from GoldBio; antibodies against hemagglutinin and ERK1/2 were from Cell Signaling Technology; antibodies against Gγ3 were from Abcam. All other materials were obtained as described (52, 53).

### Plasmid DNA constructs

The YFP-tagged Gγ plasmids (Gγ1 - #36101; Gγ2 - #36102; Gγ3 - #36103; Gγ4 - #36104; Gγ5 - 36044; Gγ7 - #36105; Gγ8 - #36106; Gγ9 - #36107; Gγ10 - #36108; Gγ12 - #36109, and Gγ13 - #36110), FLAG-tagged p110γ (# 20574), and FLAG-tagged p101 (# 20576) were obtained from Addgene. YFP-tagged Gγ11 plasmid was directly from Dr Narasimhan Gautam as described (7). The plasmids Golgi-FKBP, PM-FKBP, FRB-Gγ2, and Golgi-GRK2ct were kindly provided by Drs Alan V. Smrcka and Philip B. Wedegaertner as described (11, 14). The plasmids venus-Gβ1 and mCherry-Gαi1 were generated as described (54, 55). YFP- and DsRed-tagged p110γ and p110 were generated by using pEGFP-C1 and pDsRed-Monomer-C1 vectors, respectively. The constructs FRB-Gγ3 and FRB-Gγ9 were generated by mutating Cys in the CAAX motif of Gγ3 and Gγ9 into Ser, which were then fused with FRB. The Golgi-GRK2ctR587Q mutant was generated by using the QuikChange site-directed mutagenesis kit (Agilent). To generate rescue plasmids, two primers (5’-CATCACGCCCAAGACTTATCAGAAAAAGATTTGTTAAAGATGGAG-3’ and 5’-CTCCATCTTTAACAAATCTTTTTCTGATAAGTCTTAGGCGTGATG-3’) were used in the mutagenesis reactions using YFP-tagged Gγ9 as a template. G, T, C, C, A, G, C, G, G, C, and C at the positions 9, 12, 13, 15, 16, 17, 18, 21, 24, 27, and 28 in the nucleotide sequence of the construct Gγ9 were mutated to A, C, T, A, T, C, A, A, A, T, and T, respectively, to achieve sgRNA resistance without changing the encoded amino acid sequence.

### Cell culture and transfection

DU145, PC3, and HEK293 cells were purchased from American Type Culture Collection. DU145 and PC3 cells were cultured in complete Roswell Park Memorial Institute (RPMI) 1640 medium (Lonza) supplemented with 2 mM L-glutamine and 10% fetal bovine serum (FBS) (Atlanta Biologicals). HEK293 cells were cultured in Dulbecco's Modified Eagle's medium with 10% FBS. The transfection was carried out using Lipofectamine 3000 (Thermo Fisher Scientific).

### Generation of knockout cell lines using the CRISPR-Cas9 genome editing technology

sgRNAs were designed using CRISPOR (https://crispor.tefor.net/). sgRNAs are 5- AGCCAGCTTGTGTCGGATAA-3 (Gγ3#1), 5-TATTGGGCAAGCACGCAAGA-3 (Gγ3#2), (5- GGAAATCAAGGAGTACGTGG-3 (Gγ9#1), 5-GGTCCTTCTCGCTGAGATCC-3) (Gγ9#2), 5-CGTGCAGCAGCGCCGTTTCG-3 (p110γ #1), and 5-GTGGGCAGCACGAACTCGAT-3 (p110γ #2), which were constructed into the lentiCRISPR v2 vector (Addgene plasmid #52961) by BsmBI (New England Biolabs) as described (56). Cells were transfected with lentiCRISPR v2 vectors as control or plasmids containing sgRNAs using Lipofectamine 3000 for 24 h and selected in puromycin at a concentration of 10 μg/ml for 48 h. Knockout of the targeted proteins was determined by Western blotting.

### siRNA-mediated depletion of p101 and p87

siRNA-mediated knockdown of p101 and p87 was carried out as described (57). The cells cultured on 6-well plates were transfected with siRNA at a concentration of 30 nM using Lipofectamine 3000 for 12 h. To study the effect of p101 and p87 knockdown on ERK1/2 activation by Golgi-Gγ9, cells were transfected with siRNA, together with Gβ1, FRB-Gγ9, and Golgi-FKBP (1 μg each). The cells were then split at a ratio of 1:2 and grown for additional 24 h before starvation and stimulation with SDF1α or rapamycin.

### Confocal microscopy

To measure Gβγ translocation, cells were cultured on 25-mm coverslips for 24 h and then transfected with individual YFP-Gγ subunits, Gβ1, Gαi1, and pmTurquoise2-Golgi. Before imaging, the cells were starved for 48 h and then stimulated with SDF1α at 1 μg/ml. The cells were imaged for the YFP and cyan fluorescent protein fluorescence signals every 5 s using a time-lapse Leica DMi8 microscope. The translocation of Gβγ to the GA in response to SDF1α stimulation was quantified by measuring the increase of total YFP signal at the GA.

To measure Golgi-GRK2ct expression, cells were transfected with Golgi-GRK2ct and its mutant for 36 h and stained with GRK2 antibodies. To verify inducible translocation of Gβγ to the PM and the GA, cells were transiently transfected with venus-Gβ1 and FRB-Gγ9, together with either Golgi-FKBP or PM-FKBP (500 ng each) for 24 h and starved for 48 h before induction with rapamycin at 1 μM for 30 min. To measure the effect of Gα subunits on Gβγ translocation to the GA, cells were transfected with venus-Gβ1, mCherry-Gαi1, FRB-Gγ9, and Golgi-FKBP (500 ng each). To study Golgi fragmentation, cells were transfected with the Golgi marker YFP-GalT and then treated with different Golgi disruptors for 40 min. To study if p110γ and p101 were expressed at the GA, cells were transfected with DsRed- or GFP-tagged p110γ and p101, together with the Golgi marker pmTurquoise2-Golgi for 24 h. In these experiments, cells were fixed with 4% paraformaldehyde for 15 min. For antibody staining, cells were permeabilized with 0.25% Triton X-100 for 5 min and blocked with normal donkey serum for 30 min. The cells were sequentially stained with primary antibodies and secondary antibodies. The images were captured using a Zeiss LSM780 confocal microscope equipped with a 63× objective.

### Measurement of ERK1/2 activation

Cells were cultured in 6-well dishes for 24 h and starved for 48 h before stimulation with SDF1α, UK14304, or rapamycin as indicated in the figure legends. After the medium was removed and the cells were washed twice with cold PBS, the cells were solubilized by the addition of 300 μl of 1X SDS gel-loading buffer. ERK1/2 activation was determined by measuring ERK1/2 phosphorylation by Western blotting as described (52, 57).

### Migration and invasion assays

Chemotactic migration of PC3 cells toward SDF1α was quantified using the Boyden migration chambers. Briefly, PC3 cells were suspended in serum-free RPMI1640 medium and 2 × 10^5^ cells (200 μl) were subjected to transwell migration assays using SDF1α at 200 ng/ml for 48 h at 37 °C. For invasion assays, the suspended cells (2 × 10^5^ cells in 200 μl) were seeded in the top insert coated with diluted Matrigel solution. The migrated and invaded cells were measured by MTT assay and calculated as described (58).

### Bioluminescent imaging of luciferase in animals

All animal studies were approved by the Institution Animal Care and Use Committee of Augusta University. CRISPR-Cas9–mediated knockout PC3 and control cells were cultured and transfected with the pQCXIB plasmid. The cells were selected with blasticidin S, and luciferase expression was confirmed by dual-luciferase reporter assays. Six-week-old male nude mice (3 groups, n = 10 for each group, Jackson Laboratories) were anaesthetized and PC3 cells expressing luciferase (1 × 10^5^ cells in 100 μl) were injected *via* the left cardiac ventricle. The tumor size was measured by bioluminescent imaging of luciferase every week using an Ami Spectral Advanced Molecular Imager after intraperitoneal injection of D-luciferin (200 μl, 15 mg/ml). The data were analyzed by AMIView software and expressed as photon flux (photons/s/cm^2^/sr).

### Statistical analysis

Comparisons across groups were evaluated using one-way ANOVA, and *p* < 0.05 was considered as statistically significant. Data are expressed as the means ± SD.

## Data availability

All data presented are available upon request from Guangyu Wu (guwu@augusta.edu).

## Conflict of interest

The authors declare that they have no conflicts of interest with the contents of this article.
