# Supplementary material for: G protein βγ translocation to the Golgi apparatus activates MAPK via p110γ-p101 heterodimers
Source: J Biol Chem. 2021 Jan 23;296:100325. doi: 10.1016/j.jbc.2021.100325 (PMC7949113; doi:10.1016/j.jbc.2021.100325)
Supplement: Supplemental Figures S1–S8 [file mmc1.pdf]

Supporting information

Supplementary Figure 1

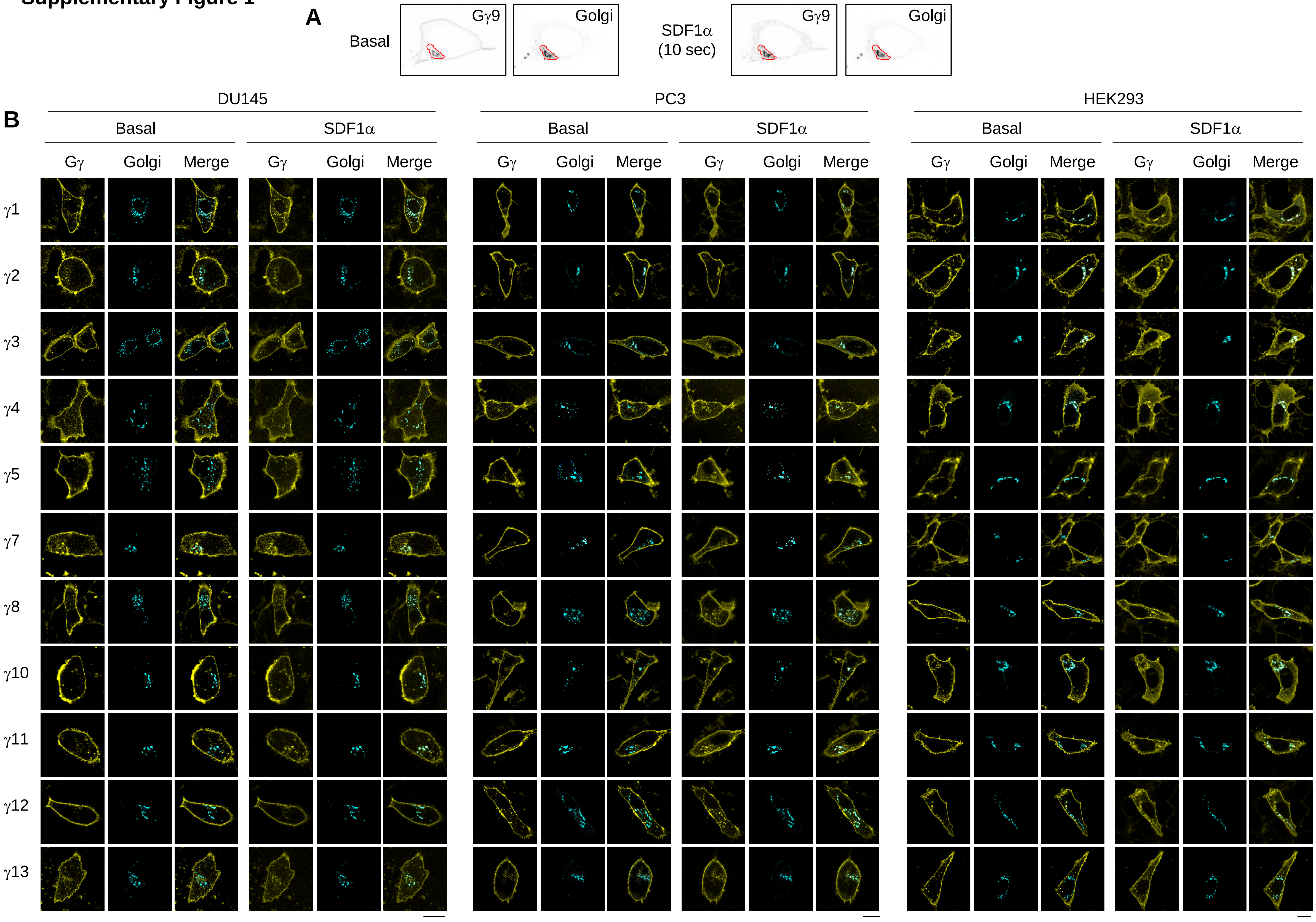

Supplementary Figure 1. Translocation of Gβγ from the PM to the GA in response to SDF1α stimulation in DU145, PC3 and HEK293 cells. (A) The quantitation of Gβγ translocation to the GA by the increase in the YFP signal at the GA in a representative DU145 cell. The GA was defined by the Golgi marker. (B) The cells were cultured on 6-well dishes and transfected with individual YFP-Gγ, Gβ1, Gαi1 and pmTurquoise2-Golgi (500 ng each). After starvation for 48 h, the cells were stimulated with SDF1α at 1 μg/ml for different time periods. The images shown are obtained after stimulation for 30, 365, 550, 235, 160, 95, 240, 215, 80, 170 and 210 sec in DU145 cells, for 30, 360, 540, 240, 155, 90, 250, 205, 85, 170 and 205 sec in PC3 cells, and for 25, 345, 530, 230, 140, 80, 25, 195, 70, 150 and 190 sec in HEK293 cells, expressing γ1, γ2, γ3, γ4, γ5, γ7, γ8, γ10, γ11, γ12 and γ13, respectively. The images are representatives of 5-8 experiments and show the maximal translocation of Gγ to the GA. Scale bars: 10 μm.

Supplementary Figure 2

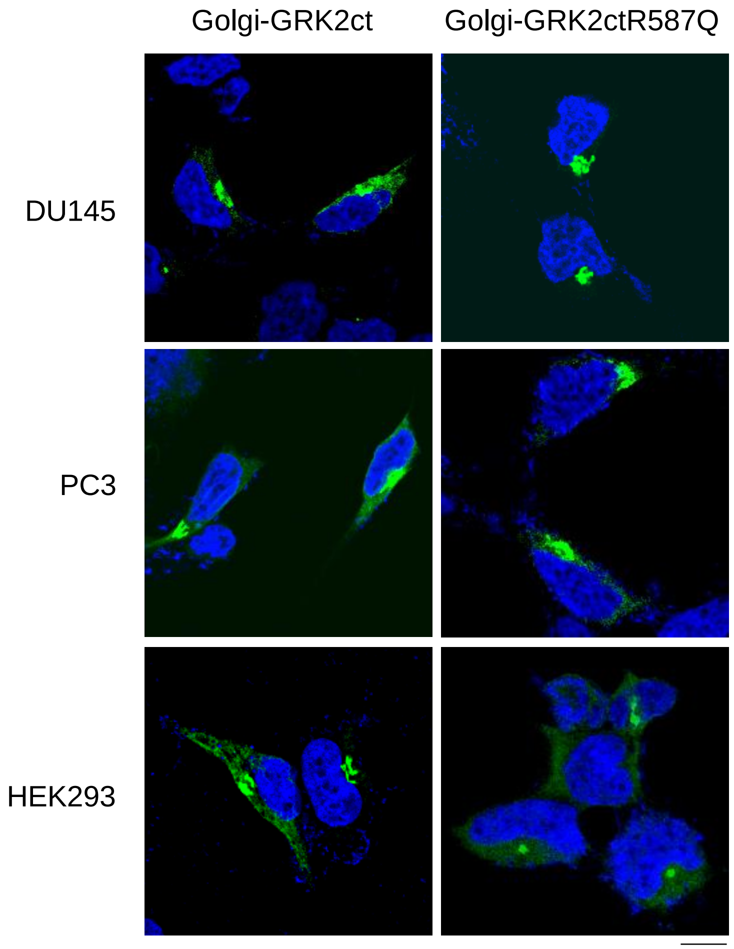

Supplementary Figure 2. Expression of Golgi-GRK2ct and Golgi-GRK2cR587Q at the Golgi. Golgi-GRK2ct and its mutant were transiently expressed in cells and their localization was revealed by confocal microscopy following staining with GRK2 antibodies. Similar results were obtained in 2 experiments. Scale bar: 10 μm.

Supplementary Figure 3

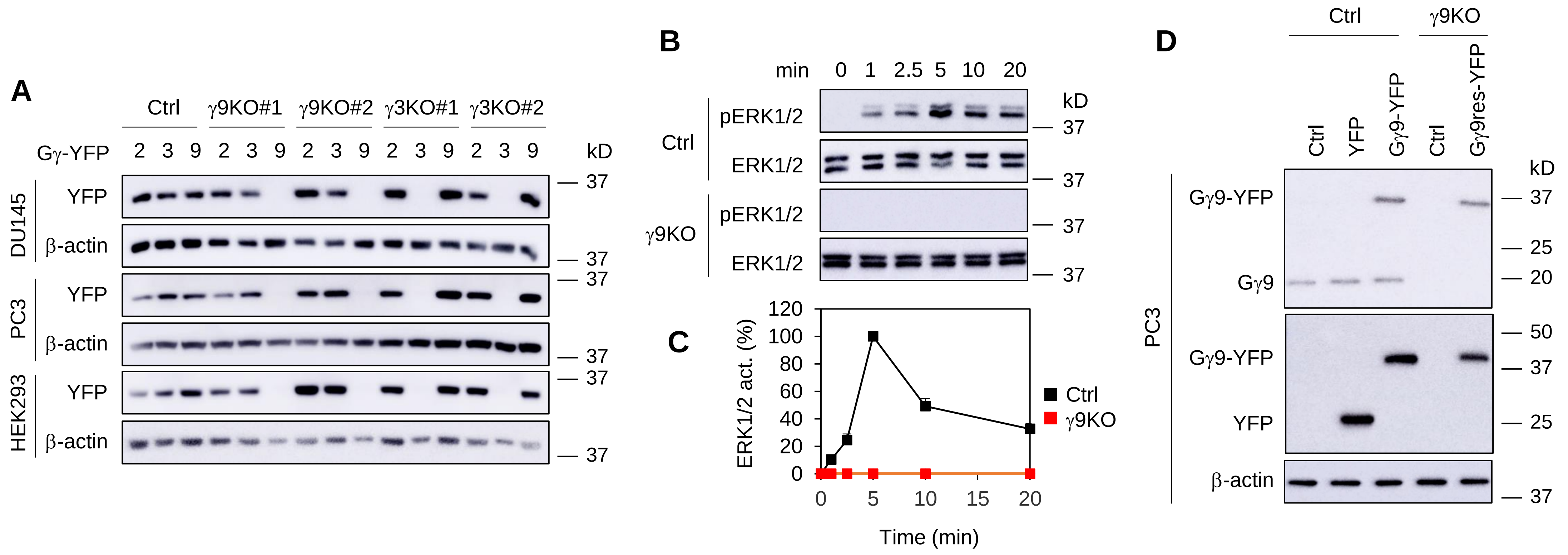

Supplementary Figure 3. CRISPR-Cas9-mediated Gγ9 knockout abolishes ERK1/2 activation. (A) Expression of YFP-tagged Gγ2, Gγ3 and Gγ9 in CRISPR-Cas9-mediated Gγ3 and Gγ9 knockout cells. The cells were cultured on 6-well dishes and transfected with 1.5 μg of individual YFP-Gγ for 48 h and YFP-Gγ expression was measured by Western blotting using YFP antibodies. (B) Time courses of ERK1/2 activation by SDF1α at 200 ng/ml in control and Gγ9 knockout PC3 cells. (C) Quantitative data shown in (B). (D) Expression of sgRNA-resistant Gγ9. Control or Gγ9 knockout PC3 cells were transiently transfected with or without YFP, YFP-tagged Gγ9, YFP-tagged sgRNA-resistant Gγ9 (Gγ9res). Expression of YFP, endogenous Gγ9 and YFP-Gγ9 was detected by immunoblotting using Gγ9 (top panel) and YFP antibodies (middle panel). Expression of β-actin was used a loading control (bottom panel). In each panel, similar results were obtained in at least 3 experiments.

Supplementary Figure 4

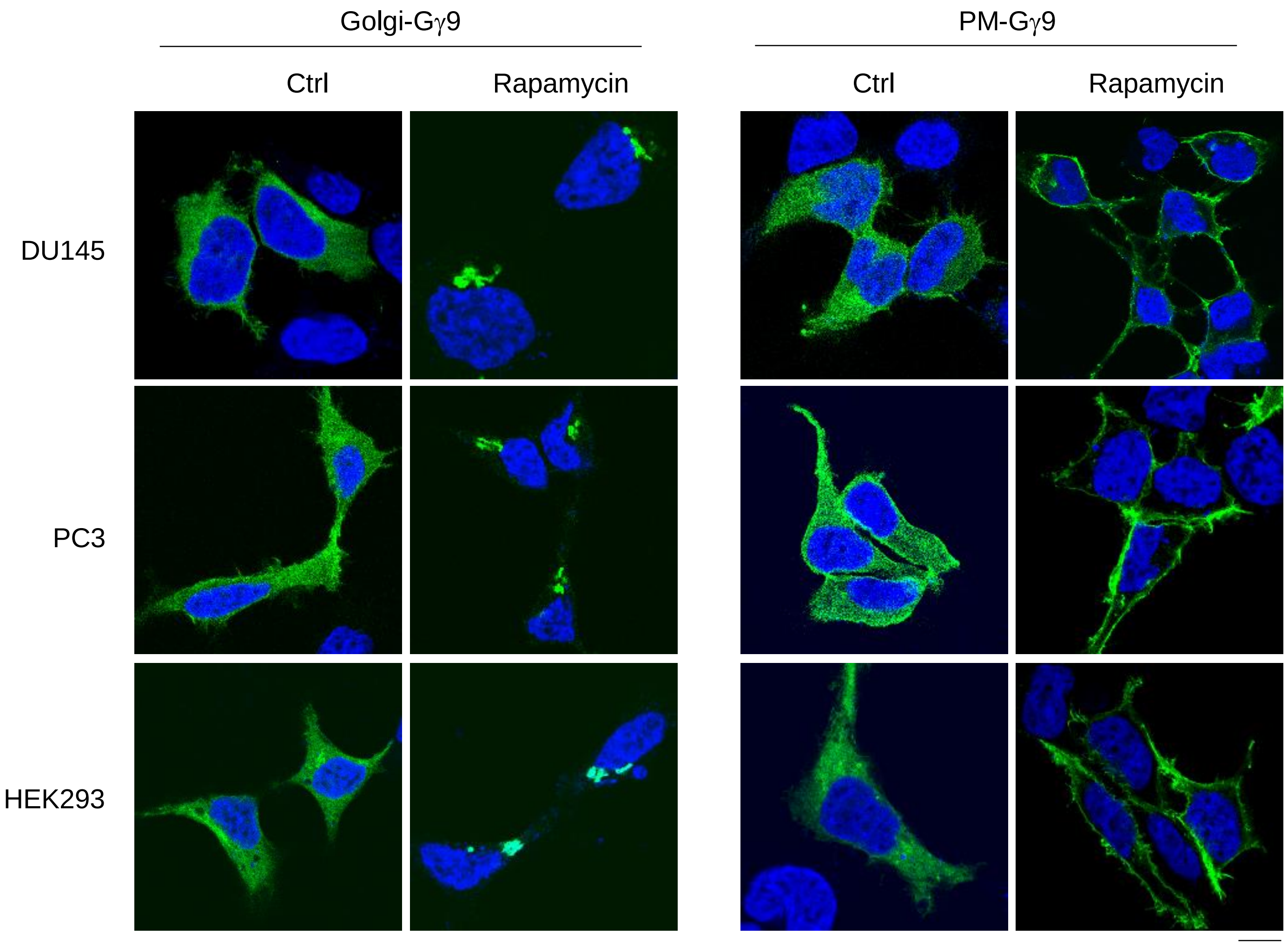

Supplementary Figure 4. Inducible translocation of Gβγ to the GA and the PM in DU145, PC3 and HEK293 cells. The cells were transiently transfected with venus-Gβ1 and FRB-Gγ9, together with either Golgi-FKBP for Golgi targeting (Golgi-Gγ9, left panel) or PM-KFBP for PM targeting (PM-Gγ9, right panel) (500 ng each), and then induced with rapamycin at 1 μM for 30 min. Gβγ translocation was revealed by confocal microscopy detecting venus-Gβ1. Similar results were obtained in 2 separate experiments. Scale bar: 10 μm.

Supplementary Figure 5

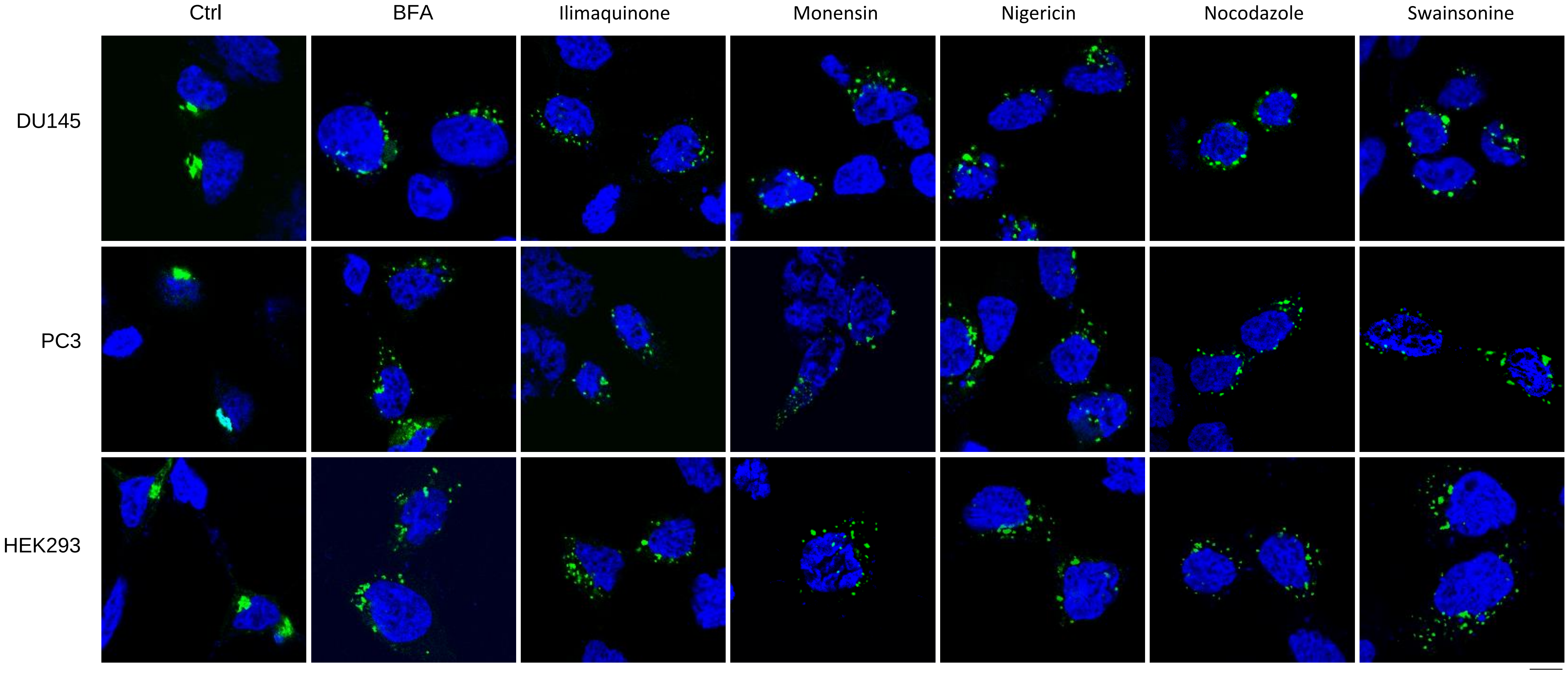

Supplementary Figure 5. Golgi fragmentation induced by Golgi disruptors. The cells were transiently transfected with YFP-GalT and then treated with BFA (3 μM), ilimaquinone (10 μM), monensin (5 μM), nigericin (2 μM), nocodazole (10 μM) and swainsonine (5 μM) for 40 min. The Golgi fragmentation was revealed by confocal microscopy. Similar results were obtained in 3 separate experiments. Scale bar: 10 μm.

Supplementary Figure 6

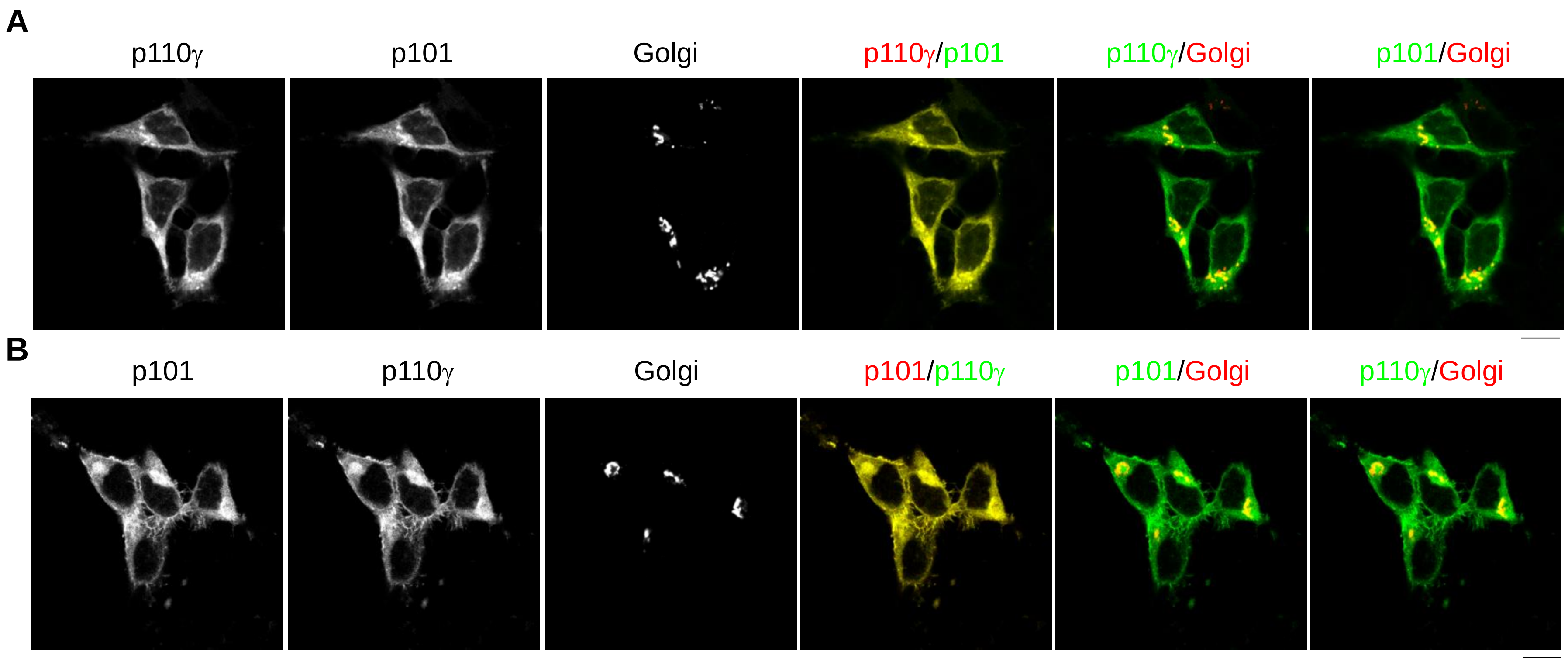

Supplementary Figure 6 Colocalization of p110γ and p101 subunits of PI3Kγ with the Golgi marker. (A) PC3 cells were transfected with DsRed-tagged p110γ, GFP-tagged p101 and the Golgi marker pmTurquoise2-Golgi. (B) PC3 cells were transfected with DsRed-tagged p101, GFP-tagged p110γ and pmTurquoise2-Golgi. Their subcellular localization was revealed by confocal microscopy.
Similar results were obtained in 3 separate experiments. Scale bars: 10 μm.

Supplementary Figure 7

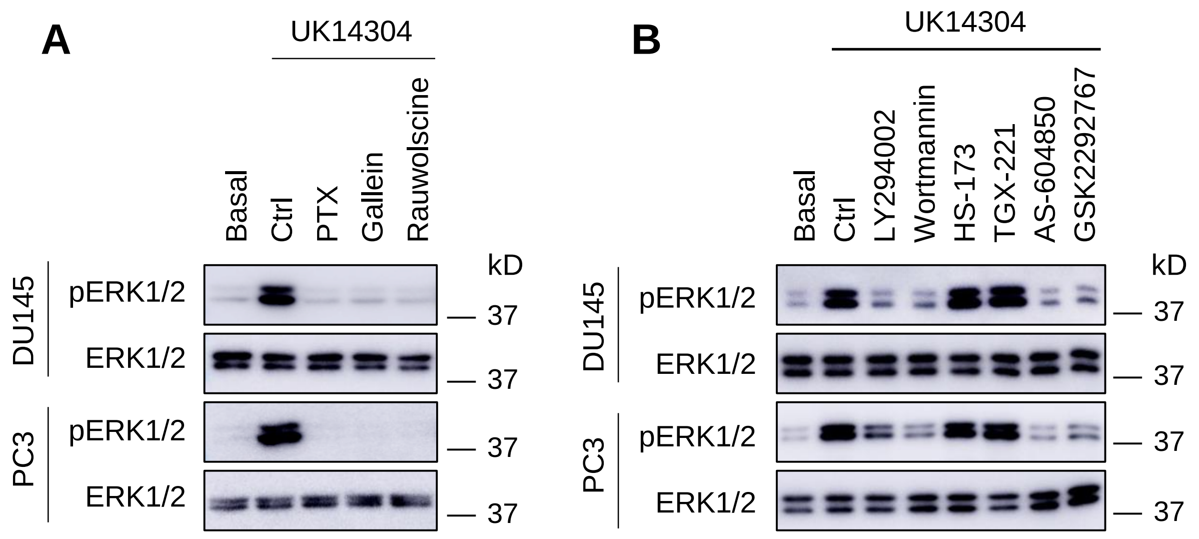

Supplementary Figure 7. Role of Gβγ and PI3K in ERK1/2 activation by α_2-AR. (A) Role of Gβγ in ERK1/2 activation by α_2-AR. The cells were treated with PTX (100 ng/ml for 16 h), gallein (10 μM for 30 min) or rauwolscine (100 μM for 1 h) before UK14304 stimulation at 1 μM for 5 min. (B) The cells were treatment with LY294002 (50 μM), wortmannin (10 μM), HS-173 (0.1 μM), TGX-221 (0.5 μM), AS-604850 (2.5 μM), GSK2292767 (0.5 μM) for 6 h before UK14304 stimulation. The Western blots shown in each panel are representatives of 3 experiments.

Supplementary Figure 8

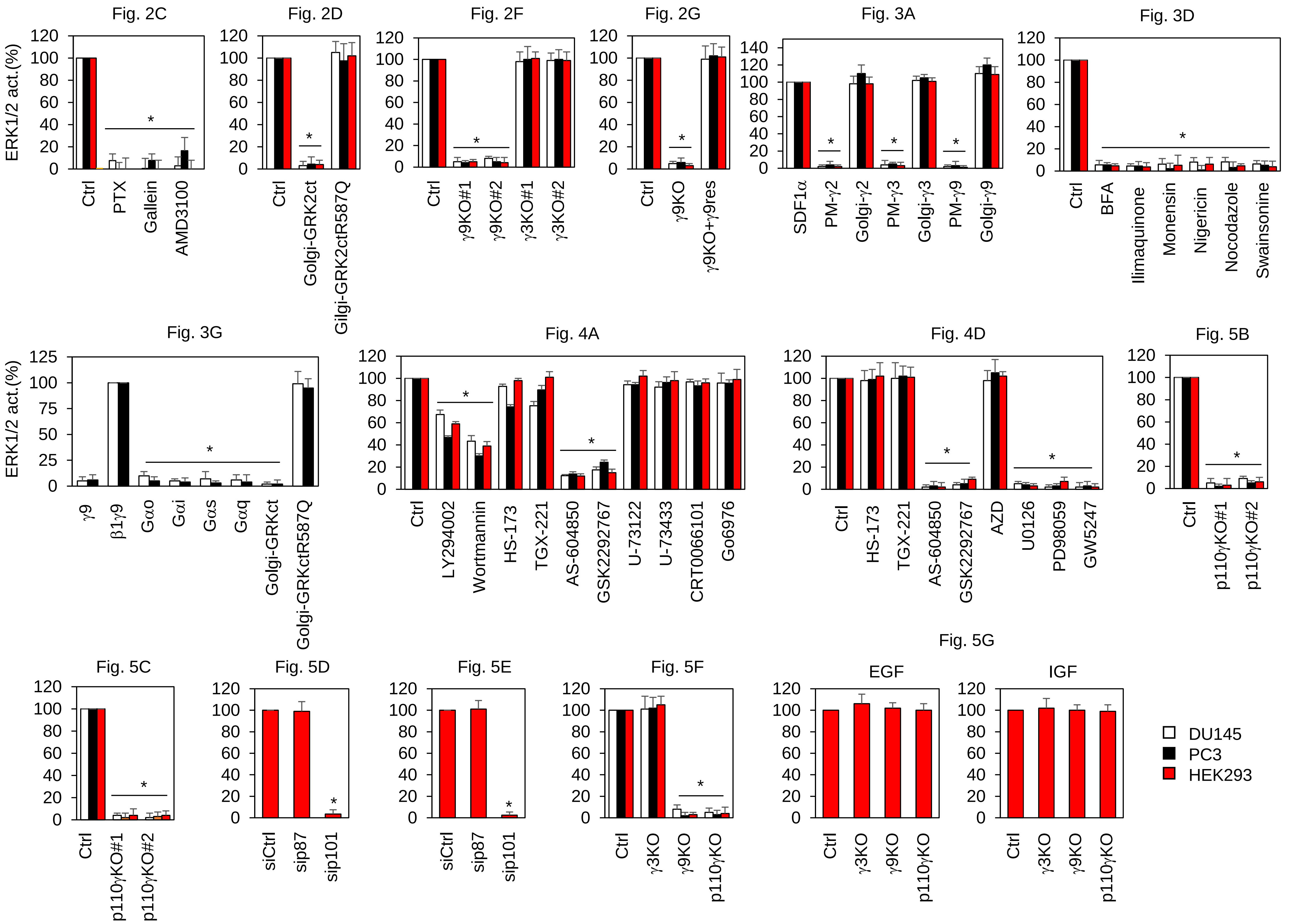

Supplementary Figure 8. Quantitative data of Western blots shown in the manuscript. *, $P < 0.05$ vs respective ctrl in Fig. 2, 3D, 4 and 5, vs SDF1α in Fig. 3A, and vs β1γ9 in Fig. 3G.
